# Spectral Color Management in Virtual Reality Scenes

**DOI:** 10.3390/s20195658

**Published:** 2020-10-03

**Authors:** Francisco Díaz-Barrancas, Halina Cwierz, Pedro J. Pardo, Ángel Luis Pérez, María Isabel Suero

**Affiliations:** 1 Department of Computer and Network Systems Engineering, University of Extremadura, E06800 Mérida, Spain; hccwierz@unex.es (H.C.); pjpardo@unex.es (P.J.P.); 2 Department of Physics, University of Extremadura, E06071 Badajoz, Spain; aluis@unex.es (Á.L.P.); suero@unex.es (M.I.S.)

**Keywords:** virtual reality, hyperspectral textures, Ishihara test, color fidelity

## Abstract

Virtual reality has reached a great maturity in recent years. However, the quality of its visual appearance still leaves room for improvement. One of the most difficult features to represent in real-time 3D rendered virtual scenes is color fidelity, since there are many factors influencing the faithful reproduction of color. In this paper we introduce a method for improving color fidelity in virtual reality systems based in real-time 3D rendering systems. We developed a color management system for 3D rendered scenes divided into two levels. At the first level, color management is applied only to light sources defined inside the virtual scene. At the second level, we applied spectral techniques over the hyperspectral textures of 3D objects to obtain a higher degree of color fidelity. To illustrate the application of this color management method, we simulated a virtual version of the Ishihara test for color blindness deficiency detection.

## 1. Introduction

Technology is constantly evolving, offering possibilities that were previously unthinkable. This is the case for image sensors that allow us to capture the three spatial dimensions and the temporal dimension of the real world and virtually represent that real world through digital images, digital videos, and, in recent years, virtual and augmented reality devices. In all cases, color is an essential part of the virtual representation of the world using digital media. This is because the recipient of that representation is a human being. For humans, color is a fundamental part of the external information they receive from their environment through their senses. The sense of sight provides more than 80% of the information received by the brain through the senses [1].

The color information captured by imaging devices often requires processing techniques to ensure correct color reproduction on various digital devices. First, it is necessary to carry out a correct chromatic characterization of both the capture device and the reproduction device. In this way, it is possible to establish a biunivocal correspondence between the device-dependent color coordinates (typically RGB or CMYK) and the device-independent color coordinates (CIE XYZ or CIE Lab). Over the last few years, a multitude of studies have been carried out on the color characterization of devices, from CRTS and TFT technology to Near-Eye Displays [2,3,4,5].

The differences in the calibration and colorimetric characterization of a color display device are always confusing [6]. Calibration of these devices consists of setting the device’s state to a known value. This can be done by fixing the media’s white point, the gain, and the offset of each channel for a cathode ray tube, for example. This process ensures that the device produces consistent results and that the calibration process can be completed without any information on the relationship between the device’s input coordinates and the colorimetric coordinates of the output. The colorimetric characterization of the device, however, requires this relationship to be known. From this characterization, the relationship between the device’s input coordinates (typically RGB values in displays and other device-independent coordinates (i.e., the CIE 1931 XYZ tristimulus value)) are obtained. Presently, all display devices are digital, and the relation between digital and analog values is accomplished through a Digital to Analog Converter (DAC). Due to the large number of chromatic stimuli that can be shown by any digital device, the direct measurement of this relation is impossible; therefore, a mathematical model is applied, enabling one to reduce the number of runs.

In addition, it is necessary to implement color management systems that allow the exchange of chromatic information between different types of devices, such as displays and printers, thereby adapting the chromatic information based on different reproduction media, different white point values, and different gamut values. Based on this need, a wide field of study of gamut mapping has been developed and has had great relevance in recent years [7]. Recently, research has been done on the color appearance in optical see-through augmented reality devices [8].

All these developments were designed primarily for developing digital images from real scenes captured by photographic techniques. The generation of synthetic digital images from 3D models, which is commonly known as 3D rendering, has been left out of this type of color management technique since color, from beginning to end, has always been defined by native digital values such as RGB values. The only color correction currently carried out is the calibration of color player devices to a standard configuration that allows a similar appearance on all displays. However, 3D development environments used in virtual reality have great versatility and computing power thanks to the GPUs that are used to render at least 90 images per second for each eye [9,10].

**Figure 1:**
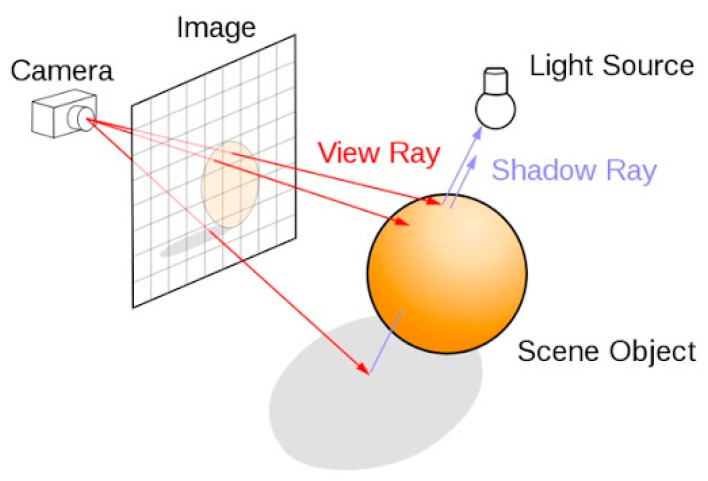
Simplified graphic representation of a lighting and shadowing method for rendering a 3D scene and how the final 2D image is generated [12].

There is a large difference between the digital images derived from photographic techniques and the digital images from 3D rendering scenes. In the first case, the image is captured by a sensor, typically CCD or CMOS, located in the image plane of the camera’s optical system, where photons arrive from different parts of the scene. In the case of a digital image obtained by rendering a 3D scene (Figure 1), a process of ray tracing is carried out in the opposite direction to that of traditional optical systems, i.e., from the eye or the camera to the objects constituting the scene, passing through a matrix of points that correspond to the future pixels [11].

From a visual point of view, Virtual Reality (VR) technology is based on the generation of two different images corresponding to two different views of the same three-dimensional scene. One of these images is shown to each eye, thereby covering a large field-of-view (FOV) and thus producing a stereoscopic image and a feeling of depth. The particularity of this stereoscopic view is that it is generated (“rendered”, in the specific language of graphic computing) while considering the position of the observer in real time, with a minimum delay and a high refresh rate of approximately 90 Hz. The position of the head and the body of the user of the VR device are continuously calculated through multiple sensors in such a way that the view of the scene corresponds exactly to the position of the observer. In this way, it is possible to create the visual sensation of immersion in a three-dimensional virtual world [13].

Large multinational companies are introducing virtual reality devices to the consumer market based on Head Mounted Displays (HMDs) with two different types of hardware: devices that do not have their own graphic hardware and need a personal computer for the task [14,15] and others that use a mobile phone or other specific graphic hardware without a personal computer [16,17]. There are significant differences in performance between both types of devices. In this work, we refer exclusively to the first type—devices associated with a personal computer with a dedicated graphics card.

**Figure 2:**
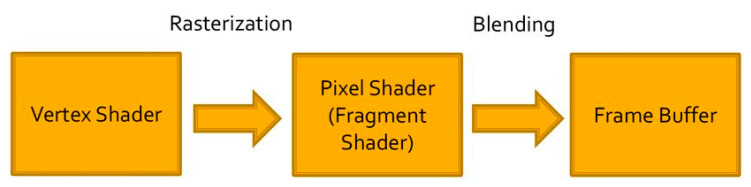
Sequence of transformations made in a Graphic Processing Unit (GPU) to generate the final 2D images for each display.

The virtual world generated in VR devices is programmable and can be created in the image and likeness of the real world or not, as needed. Currently, there are two main software platforms for developing virtual reality content: Unreal Engine [18] and Unity Game Engine [19]. In both platforms, mathematical functions are used as basic rules of internal functioning that seek to reflect, to a greater or lesser extent, the real world through physical laws [20]. The geometry of the scene is supplied to the graphics card, and this hardware then projects the geometry and breaks it down into vertices (Figure 2). Then, the vertices are transformed and split into pixels, which obtain a final rendering treatment before they are passed to the screen through the Frame Buffer.

To handle the lighting and shading conditions, the graphic engine usually uses Physically-Based Rendering algorithms that apply a bidirectional reflectance distribution function model (BRDF) with four main components (diffuse, specular, normal, and smoothness). The diffuse component corresponds to the material color with a perfect diffuse illumination, the specular component corresponds to surface color, and the normal and smoothness components both correspond to surface texture. It is, therefore, possible to obtain rendered scenes with a high degree of visual fidelity when treating the light–matter interactions this way [21,22].

Physics-based representation (PBR) includes a combination of artwork, physical properties, and material shaders that work together to give consistency to graphic representation. Using the underlying physical principles of how light and surfaces interact, we can create images that work in all lighting conditions without special cases. The combination of the high computational power of current GPU-based virtual reality systems and the use of a physically-based rendering engine for lighting and shadowing a 3D virtual scene gives us the opportunity to determine if it is possible to obtain reliable color reproduction on these types of systems by comparing a real and virtual scene. The starting hypothesis of this work is that it is possible to introduce improvements in the color reproduction fidelity of real-time 3D rendered scenes in virtual reality systems. Therefore, we propose this goal as the main objective of this work. Notably, the key question of this objective is whether the improvement of color fidelity can be done in a real-time 3D rendered scene over lights and 3D objects and not over the final 2D images sent to an HMD. To do so, we will use two different levels of color fidelity: the first one based on the color management only of light sources defined in the 3D scene, retaining the color textures of the 3D virtual objects, and the second one using spectral rendering from hyperspectral textures associated with 3D objects. The secondary goal of this work is to determine if the first level of color management is sufficient to obtain a reliable reproduction or if it is necessary to implement both levels. To illustrate the operation of this color management system applied to VR, we will create a virtual version of the famous *Ishihara Test for Colorblindness* that will help us assess the fidelity of the reproduction of a real test in a virtual scene.

## 2. Materials and Methods

The technical equipment used in this work is comprised of an *HTC Vive* HMD driven by a custom-made PC with an i7 processor (Intel, Santa Clara, CA, USA), 16 GB RAM memory, and a GeForce GTX 2060 super graphic card (Nvidia Corporation, Santa Clara, CA, USA) using the Windows 10 operating system (Microsoft, Redmond, WA, USA). The measurement instrument employed in this work was a CS-2000 tele-spectroradiometer (Konica-Minolta, Tokyo, Japan) with a spectral resolution of 1 nm between 380 and 780 nm, a <2% radiance measurement error, and CIE 1931 x = 0.0015; y = 0.0010 color error for illuminant A.

The methodology used in this work can be divided into two steps: (1) Colorimetric characterization of the VR display and (2) implementation of a color management system adapted to VR. Each step is explained in detail below.

### 2.1. Chromatic Characterization of a VR Device

The first step to use a virtual reality system in tasks related to color vision research is the chromatic characterization of the Head Mounted Display (HMD). Each device of this type has its own specific characteristics in terms of the chromaticity of its primary colors and its medium white point, as well as the relationship between the digital values of the analog-to-digital converter (DAC) and the associated tristimulus values of XYZ.

To chromatically characterize the VR device used in this study, spectroradiometric measurements were made with a tele-spectroradiometer aligned with the optical axis of the lenses with which the HMD is equipped. We measured over the display and lens assembly as a whole, leaving the measurement of the screen with and without lenses at different points of the screen for future studies. These lenses allow the user to correctly position their eyes on the displays and obtain an image from the displays with a large visual field. On the negative side, there is an increase in the image size of the pixels that makes those pixels perceptible to users. The values of chromaticity and the average relative luminance of both displays are shown in Table 1.

**Figure 3:**
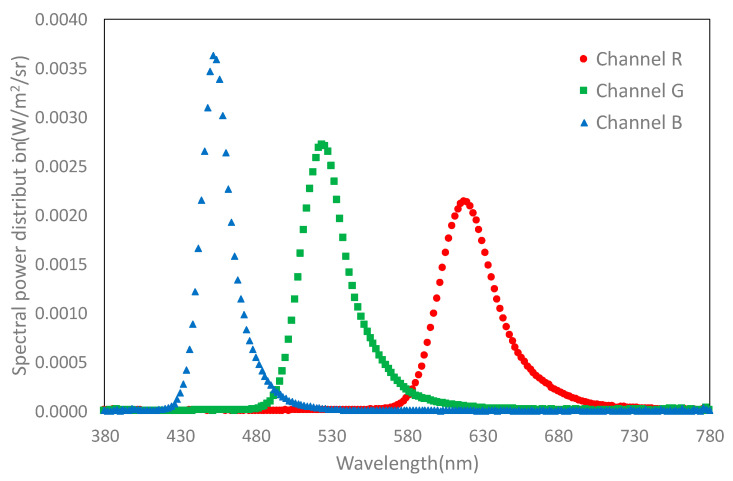
Spectral power distribution of the RGB channels measured at the maximum digital-to-analog converter (DAC) value.

The measured spectral power distribution of the RGB primaries is shown in Figure 3. The spectral radiance of each channel reveals the OLED nature of these displays with a narrow bandwidth for each channel’s RGB.

**Figure 4:**
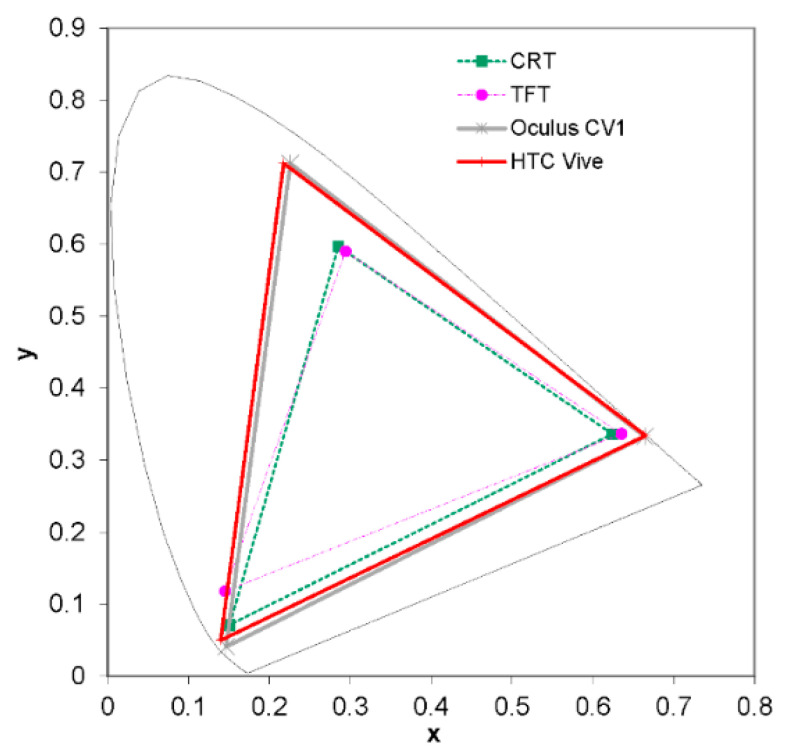
Color gamut of HTC Vive displays compared to the color gamut of different types of displays.

The color gamut is a subset of colors that can be accurately represented in a given color space or by a certain output device like a display. In this work, we measured the color gamut of our HTC Vive device by comparing its color gamut with that of other devices, such as an Oculus Rift CV1 and classic CRT and TFT monitors (Figure 4).

**Figure 5:**
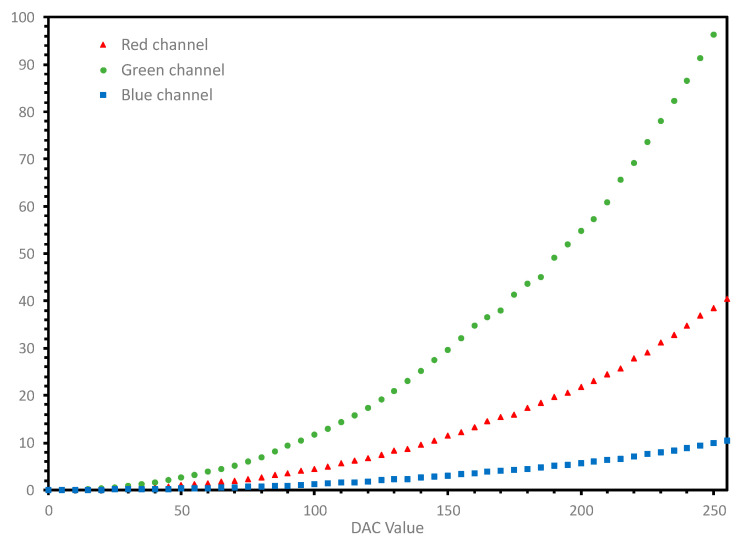
Relation between the DAC and luminance values for each RGB-independent channel.

We analyzed the relationship between the values of the digital-to-analog converter (DAC) of each RGB channel and their corresponding values of luminance Y (Figure 5). The measurements were made using our tele-spectroradiometer for each of the R, G, and B chromatic channels independently, with a range of DAC values from 0 to 255 and a step of five units.

As a result of this analysis and considering the computational time constraints of VR systems, a linear chromatic characterization model preceded by a gamma linearization stage was used. This simplified color characterization model is widely used in color management [23].

(1) $$R^{\prime }=R^{\gamma }\;G^{\prime }=G^{\gamma }\;B^{\prime }=B^{\gamma }$$

(2) $$\left(\begin{array}{c} X\\ Y\\ Z \end{array}\right)=\left(\begin{array}{ccc} X_{R^{\prime }max} & X_{G^{\prime }max} & X_{B^{\prime }max}\\ Y_{R^{\prime }max} & Y_{G^{\prime }max} & Y_{B^{\prime }max}\\ Z_{R^{\prime }max} & Z_{B^{\prime }max} & X_{B^{\prime }max} \end{array}\right)*\left(\begin{array}{c} R^{\prime }\\ G^{\prime }\\ B^{\prime } \end{array}\right)$$

This model uses a typical linear transformation between the RGB’ values and the normalized *XYZ* tristimulus values with a 3 × 3 matrix (Equation (2)). The RGB’ values were obtained after a gamma correction of the normalized RGB values that guaranteed the linearity of the system (Equation (1)). Table 1 shows the gamma value of each RGB channel and the statistical measurement of the R^2^ fit index.

To confirm the goodness of the color characterization model, we measured 50 random RGB color samples. These values were compared to those predicted by the mathematical model, thereby obtaining an average color difference of ∆E^00^ = 1.8. All measurement data together with the MATLAB script used to obtain the chromatic characterization model are available as Supplementary Material for this publication.

### 2.2. Implementation of a Color Management Procedure Adapted to VR Systems

After performing the spectral characterization of the HMD, the next necessary step to obtain a faithful reproduction of the color inside a Virtual Reality system is to introduce a color management procedure into the 3D graphics engine. The different combinations of lighting configurations in the 3D software used are practically unlimited; for example, it is possible to program different ways to perform the rendering using different shaders. The final appearance of the virtual reality scene will depend on the color of the light source used, the color of the material, the gloss, and the interaction of the different elements that form the virtual scene with shading, primary and secondary reflections, etc. For all these reasons, we assumed a series of simplifications that allow us to deal with the problem:

- We focus on the color matter, disregarding the participation of glossy objects and deactivating the secondary reflections.
- We limited the 3D software processing to real time processing, disabling the Baked and Global illumination options.
- We used Unity’s standard shader and configured the player using its linear option with forward rendering activated.

By selecting these options, we aimed to establish a configuration to analyze and compare the results of the implemented color management system.

3D scene rendering engines do not use any default color management systems. The native format used to define both light sources and object textures in this type of software is sRGB digital color space with a bit depth of 8 bits per channel. This sRGB space is widely used in computer science and image processing and is characterized by a specific gamut, defined by the chromaticity of the primaries and by a non-linear transformation (gamma) of approximately 2.2. The media White Point of this color space is D65.

The color management procedure implemented in this work has two levels of accuracy. In the first level, a C# script was implemented, which allowed to calculate the RGB values of a simulated light source in the VR scene starting from the spectral power distribution of the source and the spectral characterization of the HMD used.

The second level of precision requires the introduction of the spectral texture of the virtual objects present in the virtual scene. For this part, we developed a C# script that performs all the computational processing of the virtual object texture, thereby generating a different RGB texture for each lighting change. Notably, although in virtual reality systems the rendering is performed with a minimum frequency of 90 Hz, this rendering is done with the same light sources and RGB textures, unless they are changed at run time.

**Figure 6:**
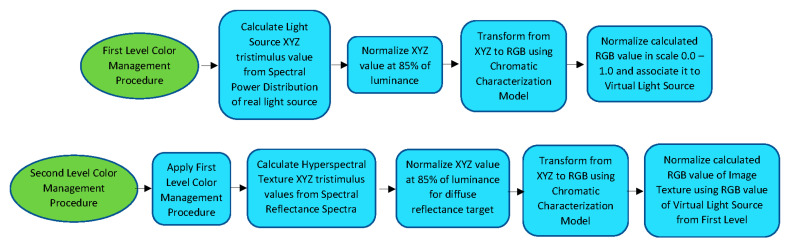
Flux diagram for both levels of the Color Management Procedure.

Figure 6 shows a flux diagram for both levels of the color management procedure developed. The first level is only applied to the virtual light sources defined inside the virtual scene. The second level requires one to apply the first one and calculate the image texture of each 3D object starting from its hyperspectral image. The entire C# script for both procedures is attached as Supplementary Material for this paper.

To analyze the results obtained by introducing both levels of color management, we used a sample of the ColorChecker color chart (X-rite, USA). This color chart is widely used in color management tasks in both scientific and professional fields. The manufacturer of this color chart provides the sRGB reference values that the color patches must present under D65 lighting. These values are presented in the first three columns of Table 2. The ColorChecker was scanned by a 3D color scanner, which provided the geometry of the object, as well as the color texture. The geometry is defined in an OBJ file as a dot-cloud. The color texture is defined by an 8-bit per channel BMP color file obtained under a D50 LED light source that the scanner is equipped with.

Our main objective is to compare the efficiency of different color management methods in real-time 3D virtual environments, such as VR. The great challenge of this work is to perform the rendering in real time, thereby solving the computational complexities that exist. To carry out this comparative study, we require not only the geometry and color texture of the ColorChecker but also the hyperspectral texture—that is, the spectral reflectance of each point of the color chart defined in the color texture file. Since this object is flat, we obtained the hyperspectral texture via a hyperspectral camera, model UHD 285 (CubertGmbH, Ulm, Germany). In this way, we replaced the RGB color texture obtained from the 3D scanner with a hyperspectral texture defined between 400 and 1000 nm, using the 4 nm steps provided by the hyperspectral camera. Starting from this hyperspectral texture file, we calculated the average RGB values of each color patch of ColorChecker corresponding to the D65 illuminant and sRGB color space. We then compared these calculated RGB values with the theorical RGB values provided by the manufacturer. Table 2 shows the reference values specified by the manufacturer and the values obtained in our calculations.

**Figure 7:**
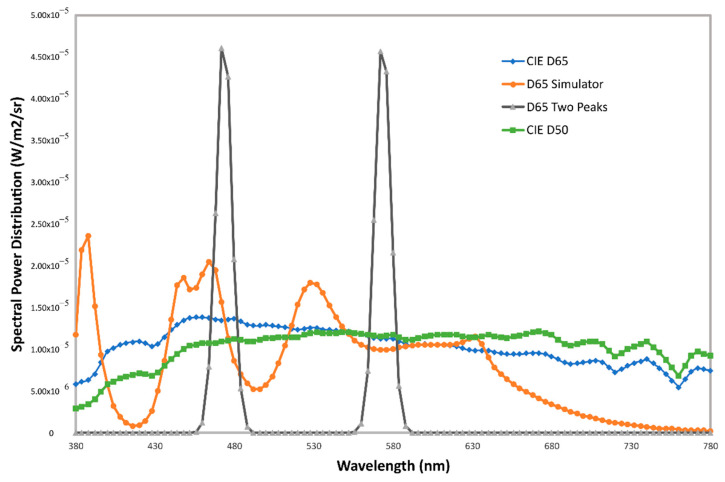
Spectral power distribution of the light sources employed in this work adjusted to a luminance value of 85 Cd/m^2^.

To study the effects of these two levels of implemented color management, we used four different virtual light sources: a D65 illuminant, a D65 simulator obtained from a commercial lightbooth in our laboratory featuring 6-peak LED technology, and a theoretical LED source composed only of two spectral peaks chosen in such a way that the color of this source over a diffuse reflectance target coincides exactly with the color of the D65 illuminant. Figure 7 shows the spectral power distribution of all light sources employed in this work.

Our 3D Graphics Engine uses sRGB as the native color space, and this space uses a medium-white-point, corresponding to the D65 illuminant. The CIE 1931 XYZ tristimulus values = (95.047 100.0 108.88) of this illuminant correspond to the digital 8-bit RGB values = (255, 255, 255). To prevent the incorrect definition of any light source whose X or Z values are above those corresponding to the illuminant D65, we chose to work with normalized sources whose relative luminance would be 85% that of the Illuminant D65. Therefore, we define the custom Illuminant D65 source as XYZ = (80.82, 85.00, 92.54), which corresponds to an RGB value = (237, 237, 237). Table 3 shows the XYZ and RGB values of the virtual light sources used, as well as their chromaticity values and Correlated Color Temperature (CCT).

The color of the objects present in a scene depends on the objects themselves but also on the light source illuminating them. The quality of a light source in terms of the fidelity of the colors it generates in a scene compared to those obtained by illuminating the scene with a reference light source can be calculated using the Color Fidelity Index R_f_, defined by the International Lighting Commission (CIE) [24]. This value is included in Table 3, which describes the characteristics of the light sources used.

**Figure 8:**
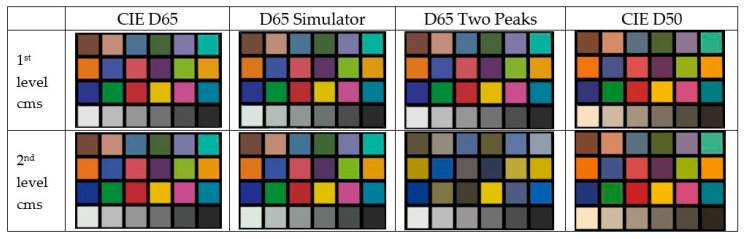
Screen capture of ColorChecker shown in the VR graphics engine with two levels of color management applied to four light sources: (first row) only applying changes in the color of the light sources and (second row) applying changes in the color of the light sources and recalculating the texture color of the VR object.

To assess the efficiency of the implemented color management system, a scene was designed in our 3D graphics engine, where only the virtual ColorChecker is illuminated by a single directional light source. The RGB values shown in Table 2 for different virtual light sources were assigned to this directional source to simulate this aspect of the ColorChecker under different light sources. In this way, the effectiveness of the first level of color management implemented (first row of Figure 8) was tested. Subsequently, the second level of color management was enabled, in which the chromatic textures of the 3D objects were recalculated according to the light source used, in addition to the actions performed at the first level (second row of Figure 8).

Figure 8 shows the results obtained when applying the two levels of color management within the 3D scene equipped with a virtual ColorChecker. At first glance, it is difficult to see the difference between the two levels of color management, except in the case of the two-peak source. In this case, the color difference is evident and results from the low Color Fidelity Index of that source, which is composed of only two spectral peaks, one blue and one yellow, making it impossible for this light source to reproduce any reddish or greenish tone. To more accurately evaluate the efficiency of both levels of color management, Table 4 shows the average color differences for each RGB channel. The RGB values measured from the screenshots are compared to those theoretically calculated from the spectral reflectance of each color patch of the ColorChecker using the different light sources. These color differences were calculated on each digital RGB channel since RGB is the native value of the implemented color management system.

In all cases, an improvement in color fidelity can be observed by applying the second level of color management. However, this difference is small, except for the case of the light source composed of two spectral peaks. There is no absolute criterion that allows knowing when it is enough to apply the first level of color management and when it is necessary to apply the two levels of color management, since it will depend on the spectral power distribution of the light source used and its interaction with the spectral reflectance of the materials of the objects used in the VR scene. In this work, it is pointed out that the Color Fidelity Index can be an indicator of when a light source may require the use of the second level of color management but setting a CFI reference value requires further research.

## 3. Results

Color reproduction can be handled at different levels of exigency depending on the need for accurate color reproduction. One of the most critical cases for color accuracy is testing for detecting color vision deficiencies. To illustrate the results obtained in this work, a reduced version of the well-known Ishihara Test for color-blindness detection [25] was virtually implemented. First, a virtual lightbooth was designed using VR-compatible 3D rendering software and then hyperspectral captures of the original test were used to introduce the captures into the virtual lightbooth. We measured the color of the virtual version of the test and compared it to the color measured in the real test. Finally, the behavior of the virtual version of the Ishihara Test was validated on real users.

### 3.1. Design of the Virtual Lightbooth and Reflectance Diffuse Reference Pattern

To analyze the results obtained in this work, a virtual scene was created by simulating a real lightbooth model, *LED Color Viewing* (Just NormLicht GmbH, Weilheim an der Teck, Germany). For this purpose, the real scale of the lightbooth and the size and position of the LED light bulbs were simulated. We performed the necessary adjustments in the 3D design software to obtain the right illuminating angle of the LED spotlights, as well as the lighting range and intensity parameters. We also created a virtual diffuse reflectance standard to perform the system calibration. Since the whole color management system is based on relative colorimetry, we need an element that allows us to determine where to locate the maximum luminance value. For this virtual lightbooth, we defined the same four virtual light sources employed in the previous step using ColorChecker.

**Figure 9:**
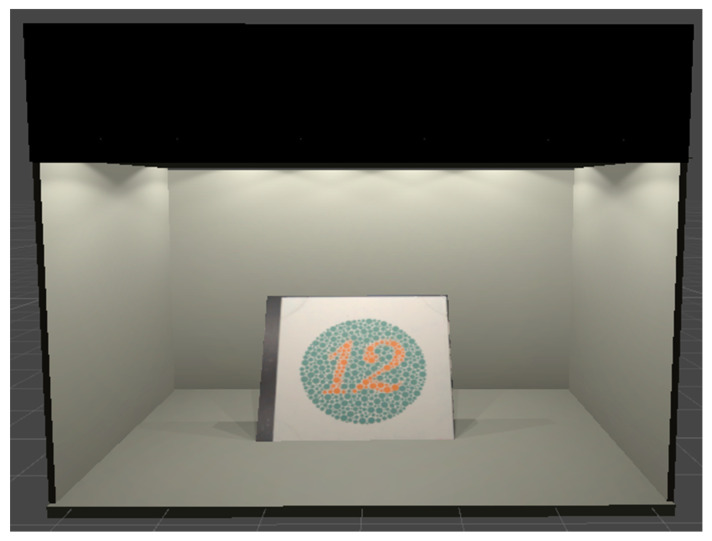
Visual appearance of the virtual lightbooth with the Ishihara test plate.

In this way, the final simulation scene was created and prepared to introduce the hyperspectral textures of the Ishihara plates. Figure 9 shows the final virtual scenario with the test plate of the Ishihara test. In this test plate, all observers must recognize the number 12.

### 3.2. Hyperspectral Textures

One of the most innovative aspects of this work is the introduction of hyperspectral textures associated with virtual 3D objects in a Virtual Reality graphics engine [26,27,28]. To illustrate this point, we created a virtual version of the Ishihara test for detecting colorblindness. In this way, we employed the database provided by the *Color Imaging Lab* of the *University of Granada* [29]. This database contains, among other elements, hyperspectral images of each plate of the Ishihara Test. The researchers of this lab also provided us a MATLAB code to process the original hyperspectral cubes corresponding to each Ishihara plate. Although the spectral range of the hyperspectral cubes is wider, the spectral range used to create the hyperspectral textures was 380–780 nm using 4 nm steps.

**Figure 10:**
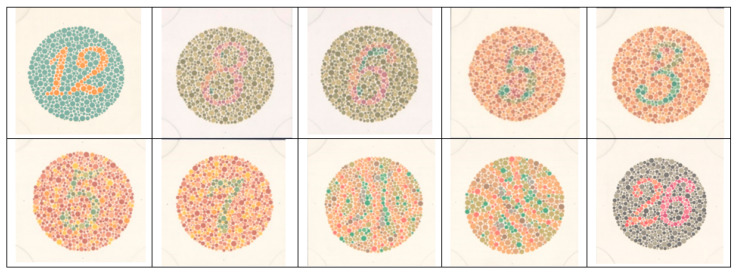
Set of 10 Ishihara plates selected to develop a quick virtual version test for colorblindness detection.

The virtual version of the Ishihara test consists of a reduced number of Ishihara plates because the only purpose of this test is to show the capabilities of this new color management system for managing hyperspectral images inside a Virtual Reality Scene with faithful color reproduction (see Figure 10). The Ishihara Test uses a reduced number of differently colored points on each slide, making it possible to easily identify the points that will be confusing to different types of defective observers based on the lines of confusion in the CIE 1931 diagram [30]. For example, to check whether correct color management was achieved in our virtual reality system and, therefore, obtain a virtual version of the original Ishihara test, we measured the 10 different colors that make up slide 3 of the virtual Ishihara test and the 10 colors that should be present, according to the colorimetric calculation from the hyperspectral images.

**Figure 11:**
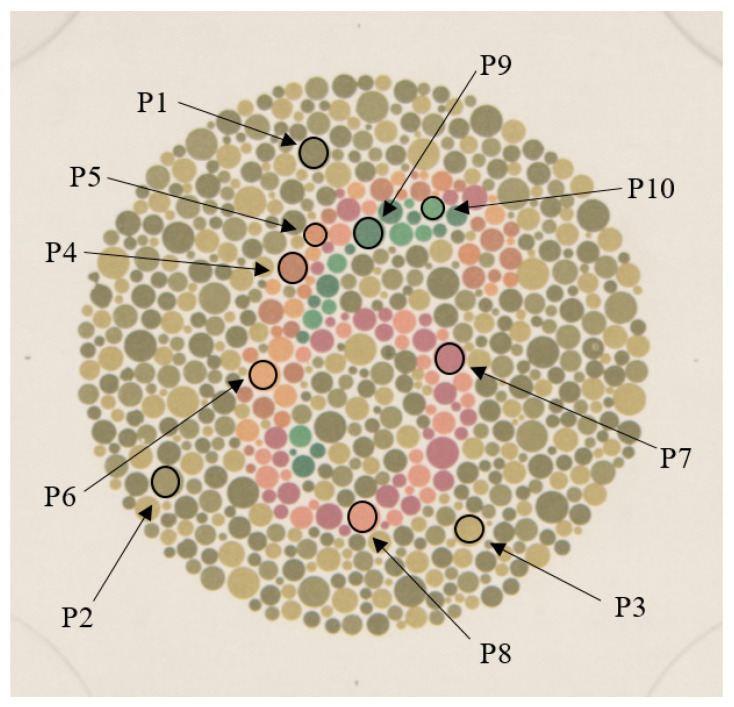
Screenshot Ishihara Plate number 3 selected to check the effectivity of color management.

Table 5 shows the calculated chromaticity of 10 different color dots of plate number 3 starting from the hyperspectral image of this plate using CIE D65 as a light source. These 10 dots belong to 10 different colors labelled from P1 to P10, as shown in Figure 11. In the same way, the measured chromaticity of the virtual reality scene for these 10 color dots is shown on the right side of Table 5. The average color difference between the calculated and measured chromaticity of these 10 samples was below 1.0 units using the CIEDE2000 color difference formula. By checking each individual difference, it can be seen that the main difference is due to luminance rather than chromaticity.

### 3.3. Validation of the Procedure of Color Management in VR

Finally, a validation of the virtual version of the rapid test for detecting color vision deficiencies based on a reduced version of the *Ishihara Test* was conducted. This test was carried out on 10 observers previously assessed in our laboratory for their color perception abilities. Of these 10 observers, six had previously been classified as normal observers using the Farnsworth–Munsell 100 Hue test and another four as defective observers (1 deutan and 3 protan). Table 6 shows the detailed responses for each plate of the virtual test, as well as the reference responses following the Ishihara Test instructions. The results obtained by the virtual test match those obtained by the real test and the previous cataloguing of the results. The purpose of this test was to validate the color management procedure carried out in the virtual reality environment via a psychophysical test, rather than to validate the test itself, which would require a greater number of tests and observers.

## 4. Conclusions

The results of this work can be measured in terms of the color reproduction fidelity obtained through the two levels of color management implemented. From this point of view, the results indicate that the application of the first level of color management, which only affects the lights and employs 3D scanned textures, may be sufficient in cases where high accuracy color reproduction is not required, provided that the spectrum of the simulated light source does not differ much from the spectrum of the reference illuminant. Under these assumptions, color differences of about 2 RGB digital units are achieved. This is not the case if the simulated light source has a low R_f_ color rendering index. In this case, it will be necessary to apply both levels of color management using the hyperspectral textures of the objects represented in the virtual reality scene. Applying these two levels of color management in VR will result in a very high color fidelity, with an average color difference below one RGB digital unit on each channel. In this case, then, the only significant color difference will be that coming from the chromatic characterization of the HMD, which will depend on the characteristics of each display used in the HMD.

The first level of color management implemented can be applied to any 3D object captured using a 3D scanner or other 3D capture technology. However, the second level requires the use of a hyperspectral camera to capture the information related to the spectral reflectance of the materials that conform the object. In the case of objects with flat geometry, such as the Ishihara test plates used in this work, their use in virtual reality environments is immediate. In the case of objects with other less simple geometry, it is necessary to use photogrammetric technics, like Multi-View 3D Reconstruction to capture a virtual 3D object from a real 3D object. This technique belongs to the category of *Structure From Motion* (SFM) technics, in which three-dimensional structures are estimated from two-dimensional image sequences. Traditionally, all these technics are based on RGB images captured by one or several digital RGB cameras. Our approach to solve this limit consists in substituting the RGB camera by a hyperspectral camera with different spectral channels. In this way we could obtain the 3D point cloud that defines the geometry of the 3D object and also, we could obtain the associated hyperspectral texture. This is a line of research we are currently working on.

Considering the above, we can conclude that the implemented color management methods can be applied in virtual reality scenes to facilitate the correct simulation of scenes where light and color fidelity are important factors. Obviously, these results can be applied to other non-VR 3D rendering systems, but the challenge was to define a color manage procedure for VR systems because of the high refresh rates of such systems.

## Figures and Tables

**Table 1:** The average and standard deviation of CIE 1931 chromaticity and the relative luminance of one point of each display of the HMD measured through the lens.

Channel	Chromaticity		Luminance	Gamma	
	x	y	Y (Relative)	Value	R^2^
White	0.299 ± 0.002	0.315 ± 0.002	100.0		
Red	0.667 ± 0.004	0.332 ± 0.003	30.3 ± 1.1	2.43	0.999
Green	0.217 ± 0.007	0.710 ± 0.002	75.4 ± 2.4	2.38	0.999
Blue	0.139 ± 0.002	0.051 ± 0.002	7.8 ± 0.5	2.38	0.998
Black	0.311 ± 0.01	0.307 ± 0.004	0.4 ± 0.2		

**Table 2:** Reference RGB values for the ColorChecker patches versus those calculated from the hyperspectral image.

ColorChecker	Reference			Measured		
Patch Number	R	G	B	R	G	B
1	115	82	68	115	82	67
2	194	150	130	195	149	129
3	98	122	157	93	123	157
4	87	108	67	90	108	65
5	133	128	177	130	129	176
6	103	189	170	99	191	171
7	214	126	44	219	123	45
8	80	91	166	72	92	168
9	193	90	99	194	85	98
10	94	60	108	91	59	105
11	157	188	64	161	189	63
12	224	163	46	229	161	41
13	56	61	150	43	62	147
14	70	148	73	72	149	72
15	175	54	60	176	49	56
16	231	199	31	238	199	24
17	187	86	149	188	84	150
18	8	133	161	0	136	166
19	243	243	242	245	245	240
20	200	200	200	200	202	201
21	160	160	160	160	161	161
22	122	122	121	120	121	121
23	85	85	85	83	84	85
24	52	52	52	50	50	50

Average difference of ΔR = 3.4, ΔG = 1.6, ΔB = 1.9.

**Table 3:** Numerical characterization of the light sources used in this work: CIE R_f,_ Color Fidelity Index; Correlated Color Temperature, CCT; CIE 1931 chromaticity coordinates and tristimulus values; and the RGB values corresponding to the sRGB standard.

Light Source	CIE Rf	CCT	CIE 1931 x,y	CIE 1931 XYZ	RGB
CIE D65	100	6503	0.3127, 0.3289	80.81, 85.00, 92.57	237, 237, 237
D65 Simulator	88.2	6568	0.3107, 0.3344	79.00, 85.00, 90.31	232, 239, 234
D65 Two Peaks	3.2	6501	0.3127, 0.3291	80.74, 85.00, 92. 53	237, 237, 237
CIE D50	100	5000	0.3458, 0.3585	81.98, 85.00, 70.11	255, 235, 205

**Table 4:** Average and standard deviation of the color differences between the calculated and measured RGB colors for the four light sources employed.

Light Source	1st Level CMS			2nd Level CMS		
	R	G	B	R	G	B
CIE D65	3.1 ± 3	1.4 ± 1	2.0 ± 2	0.2 ± 0.4	0.5 ± 0.5	0.2 ± 0.4
Simulator D65	2.6 ± 2	1.7 ± 1	3.3 ± 5	0.5 ± 0.5	0.2 ± 0.4	0.4 ± 0.6
D65 Two Peaks	30 ± 29	9.0 ± 8	6.5 ± 7	0.5 ± 0.5	0.4 ± 0.5	0.5 ± 0.5
CIE D50	3.1 ± 3	1.7 ± 2	4.6 ± 7	0.1 ± 0.3	0.4 ± 0.5	1.2 ± 0.9

**Table 5:** Chromatic coordinates calculated from the hyperspectral image and measured from the virtual reality scene for 10 different color dots of Ishihara Test plate number 3.

Color Dot	Calculated Color			Measured Color			ΔE
	x	y	Y	x	y	Y	DE2000
P1 Green Dark	0.3560	0.3854	24.6	0.3557	0.3850	25.4	1.00
P2 Green Medium	0.3602	0.3906	29.1	0.3601	0.3903	30.0	1.05
P3 Green Light	0.3734	0.3986	38.4	0.3731	0.3984	41.0	0.82
P4 Ochre Dark	0.3901	0.3656	27.1	0.3895	0.3655	28.1	0.70
P5 Ochre Medium	0.4022	0.3746	33.1	0.4014	0.3743	34.3	0.76
P6 Ochre Light	0.3945	0.3834	41.5	0.3941	0.3834	42.8	1.28
P7 Purple Dark	0.3713	0.3282	24.2	0.3709	0.3282	25.6	1.14
P8 Purple Light	0.3884	0.3576	36.1	0.3878	0.3573	38.0	0.72
P9 Bluish-Green Dark	0.3158	0.3759	23.7	0.3159	0.3763	22.7	0.64
P10 Bluish-Green Light	0.3182	0.3881	33.3	0.3182	0.3887	31.7	1.47

Average ΔE^00^ = 0.96.

**Table 6:** Numerical responses of the 10 real observers to the 10 plate virtual version of the Ishihara Test.

Observer	Qualification	P1	P2	P3	P4	P5	P6	P7	P8	P9	P10
Ref1	Normal	12	8	6	5	3	7	3	-	-	26
Ref2	Protan	12	3	5	2	5	-	-	5	8	6
Ref3	Deutan	12	3	5	2	5	-	-	5	8	2
Ob1	Normal	12	8	6	5	3	5	7	-	8	26
Ob2	Normal	12	8	6	5	3	5	7	-	-	26
Ob3	Normal	12	8	6	5	3	5	7	-	-	26
Ob4	Normal	12	8	6	5	3	5	7	-	-	26
Ob5	Normal	12	8	6	5	3	5	7	-	-	26
Ob6	Normal	12	8	6	5	3	5	7	-	-	26
Ob7	Protan	12	3	5	2	-	-	-	5	2	6
Ob8	Protan	12	3	5	2	5	-	-	5	8	6
Ob9	Deutan	12	3	5	2	-	-	-	5	8	2
Ob10	Protan	12	3	5	2	5	-	-	5	8	6

## Supplementary Materials

The following are available online at https://www.mdpi.com/1424-8220/20/19/5658/s1, Source Code S1: Calibration Script. Source Code S2: Color Management Script.
